# Emergency laparoscopic repair of left paraduodenal hernia in a young man: a case report

**DOI:** 10.3389/fsurg.2026.1902302

**Published:** 2026-07-15

**Authors:** Song Geng, Hong Jiang, Guole Nie, Longlong Geng, Tao Hao

**Affiliations:** Department of Colorectal Hernia Surgery, Shandong Medical and Pharmaceutical University Hospital, Binzhou, China

**Keywords:** case report, intestinal obstruction, intra-abdominal hernia, laparoscopic hernia repair, paraduodenal hernia

## Abstract

**Background:**

Paraduodenal hernia (PDH) isa rare congenital intra-abdominal hernia, especially in young people. It is often caused by abnormal intestinal rotation. The clinical manifestations lack specificity and are frequently misdiagnosed or overlooked. This article reports the diagnosis and treatment of a young male with left paraduodenal hernia (PDH) and explores its diagnosis and minimally invasive treatment strategy.

**Case data:**

A 26-year-old male patient was admitted to the hospital due to “abdominal pain for 7 h”. Physical examination showed tenderness in the left upper abdomen. CT revealed clustered bowel loops adjacent to the ascending duodenum, and PDH was considered. Emergency laparoscopic exploration confirmed that part of the jejunum herniated into the Landzert recess. Hernia ring release, intestinal tube reduction and hernia ring suture repair were performed, and the patient recovered well after the operation.

**Discussion:**

Preoperative diagnosis of PDHrelies on imaging, especially enhanced CT, while laparoscopic surgery enables precise reduction of herniated contents and closure of the defect, reducing postoperative complications. This case suggests that even young patients with unexplained intestinal obstruction should be alert to the possibility of PDH, and early surgical intervention can improve prognosis.

**Conclusion:**

Although rare, PDH carries significant risk of fatality. Clinicians need to raise their awareness. Combined with early diagnosis by imaging, laparoscopic surgery is a safe and effective treatment method.

## Background

Paraduodenal Hernia (PDH) is the most common congenital intra-abdominal hernia, mainly caused by abnormal midgut rotation during the embryonic period. The clinical manifestations of PDH are diverse, ranging from asymptomatic to acute intestinal obstruction and even intestinal necrosis. Preoperative diagnosis is extremely challenging and has a high misdiagnosis rate. With the advancement of imaging technology, enhanced CT has become the gold standard for the diagnosis of PDH. Surgery is the only radical cure for PDH. Traditional open surgery is increasingly supplanted by laparoscopy, which has the advantages of less trauma and faster recovery, but the indications must be strictly controlled. In recent years, the application of robot-assisted surgery and new suture materials (such as barbed wire) has further optimized the surgical effect. However, for patients with strangulated hernia or hemodynamic instability, open surgery is still the first choice. This report details the clinical presentation, imaging findings, and minimally invasive management of a young patient with PDH through the diagnosis and treatment process.

## Medical records

### General information

The patient, a 26-year-old male, was admitted to the hospital due to “abdominal pain for 7 h”. 7 h before admission, the patient suddenly developed persistent moderate pain in the upper abdomen, the pain did not radiate to other parts, accompanied by cessation of flatus and defecation, no nausea and vomiting, and then he was treated in the emergency department of our hospital. He had no history of abdominal pain and abdominal surgery.

### Investigations

T: 36.2℃, P: 92times/min, R: 23times/min, Bp: 144/101mmHg. Abdominal examination: The abdomen is flat, no gastrointestinal type, no abdominal wall varicose veins. The abdomen is soft, no abdominal mass is palpable, there is tenderness in the abdomen, especially in the left upper abdomen, and no rebound pain. Abdominal shifting dullness is negative, and bowel sounds are normal. Laboratory examination (blood routine): white blood cell count 9.6*10^9^/L, neutrophil percentage 94.8%. Whole abdominal CT enhancement: The mesentery in the middle abdomen is not natural, bowel loops appear clustered and rotated, located at the edge of the ascending duodenum, between the lower stomach and the pancreas, and there is no obvious sign of dilatation of the intestinal tube. Consider intra-abdominal hernia (left paraduodenal hernia) (Figure 1).

**Figure 1:**
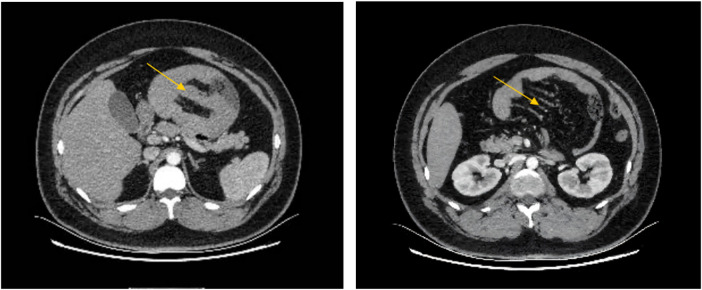
CT images demonstrate abnormal mesenteric in the middle abdomen is not natural, the intestinal tubes are relatively aggregated and rotated, and are located at the edge of the ascending duodenum.

## Treatment

After admission, the patient was given treatment such as fasting, fluid replacement, and anti-infection. After the diagnosis was confirmed, emergency surgery was decided. After successful anesthesia, the patient was in a supine position, and a small incision below the umbilicus was made to place a 10 mm diameter Trocar to establish an observation hole. Laparoscopic exploration was performed, and part of the jejunum herniated into the Landzert recess on the left side of the ascending duodenum. Membranous adhesions were seen around it, restricting the activity of the intestinal tube. During the operation, a left paraduodenal hernia was diagnosed (Figure 2A). A small incision was made on the lateral edge of the right lower abdominal rectus abdominis to place a 5 mm Trocar to establish two operation holes. The adhesion bands were sharply dissected using an electrocautery hook, free the herniation into the intestinal tube, and restore the normal anatomical structure of the intestinal tube. During the operation, about 40 cm of the jejunum was found to have herniated into the recess. The intestinal tube was slightly dilated but had good blood supply, and no perforation or necrosis was found. The Landzert recess was fully exposed, and the hernia ring was about 6*5 cm (Figure 2B). The defect was closed with a 2–0 barbed suture (Figure 2C) to prevent recurrence. There was no active bleeding during the examination, the intestinal tube was arranged naturally, and the trocar holes were closed layer by layer. The operation time was 105 min, and the amount of bleeding was about 5 mL. After the operation, symptomatic treatment such as anti-infection and nutritional support was given, and the patient's vital signs were stable. On the first day after the operation, the patient started a liquid diet after passing gas, and the abdominal pain symptoms were significantly relieved; on the second day, the patient started a semi-liquid diet after defecation; on the fourth day, the patient recovered well and was discharged. The patient returned to the hospital for a follow-up examination one month after discharge, and underwent full gastrointestinal barium series and abdominal CT examination (Figure 3B). The patient did not complain of obvious discomfort.

**Figure 2:**
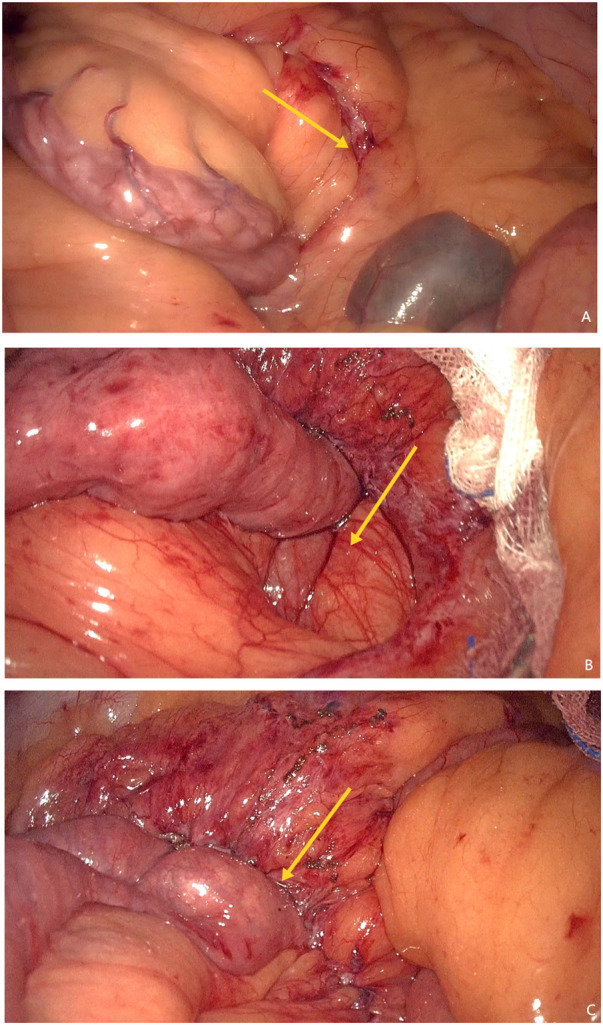
Intraoperative laparoscopic images. **(A)** small intestine herniates into Landzert's recess, **(B)** hernial ring after freeing the hernial contents, **(C)** hernial ring after closure.

**Figure 3:**
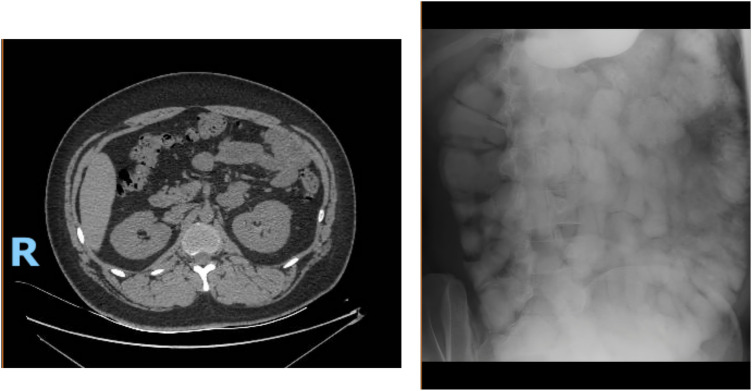
The patient's postoperative follow-up of full gastrointestinal barium series and abdominal CT.

## Patient perspective

The patient reported experiencing persistent abdominal pain in the early stages of the illness without any apparent cause, which worsened in the later stages and was accompanied by cessation of bowel movements. Realizing the severity of the condition, the patient urgently sought medical attention at the hospital. When I learned about suspected intra-abdominal hernia and the need for emergency surgery, I felt anxious and worried about the risks of surgery and postoperative recovery. Postoperative abdominal pain symptoms completely disappeared, gastrointestinal function gradually recovered, and normal dietary activities were resumed. The patient is satisfied with the surgical treatment effect.

## Discussion

Intra-abdominal hernia is a special type of hernia caused by the protrusion of abdominal organs or tissues through normal or abnormal openings in the abdominal cavity. Its characteristic is that the hernial contents remain in the abdominal cavity without breaking through the abdominal wall. According to the cause, it can be divided into two categories: congenital and acquired (1). Intra-abdominal hernia is a rare disease, accounting for less than 1% of all abdominal hernias (2), but its potential risks cannot be ignored.

Paraduodenal hernia (PDH) is the most common type of congenital intra-abdominal hernia, accounting for more than 50% of all intra-abdominal hernias. Based on the anatomical location, PDH can be divided into right-sided (RPDH) or left-sided (LPDH). Left-sided PDH is relatively more common and usually occurs in males. It has a 50% chance of developing small bowel obstruction and a mortality rate of 20%–50%. Schizas et al. reported that the average age of PDH onset is 44.1 years (3). The incidence of PDH in males is three times that in females (4). LPDH is more common in middle-aged individuals, but there have been reports of cases in adolescents. Yıldız et al. have reported cases of adolescent onset (5). There are many theories about the mechanism of paraduodenal hernia formation. The most accepted theory, first proposed in 1923, is that PDH formation is related to abnormal mid-embryonic intestinal rotation and peritoneal fixation. During embryonic weeks 6–10, the midgut (which will develop into the small intestine and part of the colon) rotates around the superior mesenteric artery and is eventually fixed to the posterior abdominal wall. Malrotation or fixation failure may result in peritoneal folds forming the Landzert recess (left side) or Waldeyer's recess (right side), becoming the anatomical basis of the hernia sac (6).

The clinical manifestations of PDH are broad, ranging from asymptomatic (incidental imaging findings) to acute intestinal obstruction. Although the symptoms of “abdominal pain + cessation of flatulence and defecation” in this case are nonspecific, the diagnosis can be quickly confirmed by combining the mesenteric “whirlpool sign” on CT. It is worth noting that PDH symptoms may progress faster in young patients—in this case, the neutrophil ratio increased (94.8%) in only 7 h from onset to surgery, suggesting the need for more aggressive intervention. The contents of PDH are usually the stomach and small intestine, and sometimes the colon. Due to the lack of specific clinical manifestations, the diagnosis of PDH remains a challenge and complicates differential diagnosis. As the disease progresses, a large number of PDH cases may develop intestinal necrosis, intestinal perforation, and even death (7, 8). For patients with recurrent abdominal pain and intermittent intestinal obstruction, the diagnosis of PDH should be considered in the absence of a history of surgery, abdominal wall hernia, and intra-abdominal inflammatory disease, especially when the inferior mesenteric vein moves forward (9). Understanding the anatomy of the mesentery, peritoneal folds, duodenal fossa, and embryology of the midgut is important for the diagnosis of PDH (10). Due to the high risk of obstruction and high mortality, modern imaging techniques should be used to make a diagnosis preoperatively. Abdominal CT is the preferred examination for diagnosing PDH. Abdominal CT may help to demonstrate displacement or tortuosity of the vessels to aid in the clinical diagnosis of PDH (11). For cases that cannot be diagnosed by imaging methods, diagnostic laparoscopy can be attempted to verify the diagnosis and synchronize surgical intervention (12).

Surgical intervention is the main treatment for PDH. For all confirmed PDH (including asymptomatic cases), PDH surgery is necessary because the risk of strangulation is as high as 15%–20% (13, 14), and early surgery can significantly reduce the mortality rate and improve the patient's prognosis (15). The treatment method of laparotomy and laparoscopy depends on the surgeon's expertise and available infrastructure (16, 17). The first case of a left paraduodenal hernia repaired entirely laparoscopically was reported in 1998 (18). Studies have shown that laparoscopic hernia repair for PDH has significant advantages over open surgery. Laparoscopic treatment of PDH can shorten hospital stay, promote early feeding, and reduce the incidence of perioperative complications, but the long-term effects of the two methods are similar (19, 20). Laparoscopic surgery also carries the risk of iatrogenic injury, including small bowel rupture due to incarcerated intestinal dilatation, abdominal congestion, and massive exudate. The incidence and preoperative diagnosis rate of PDH are low, and the timing of surgery is very important (17). However, laparotomy remains indicated for ischemia, strangulation, visceral perforation, intestinal dilatation (intestinal diameter > 4 cm), and hemodynamic instability (21). Regardless of the surgical approach, the principles of treatment for PDH include reducing the contents, resecting the hernial sac, restoring normal intestinal anatomy, and repairing the hernial ring (22). At the same time, there are also cases that emphasize the effectiveness of robotic repair of paraduodenal hernias (23). A key consideration in the surgical treatment of paraduodenal hernias is the prevention of recurrence (24). In this case, we used 2–0 barbed sutures to close the 6 ×  5 cm hernial ring. Its tensile strength can reduce the risk of recurrence, which is particularly important for young patients because of their long life expectancy and the need to ensure long-term anatomical reduction.

## Conclusion

Paraduodenal hernia (PDH) is a rare congenital intra-abdominal lesion with atypical clinical symptoms, easily leading to missed diagnosis of intestinal obstruction. The present case of a 26-year-old male patient with left PDH complicated by acute abdominal pain achieved a favorable outcome after laparoscopic surgical repair, validating the safety and feasibility of minimally invasive laparoscopy for PDH treatment. Early and accurate imaging examination is essential for definitive PDH diagnosis, and laparoscopic surgery is the preferred minimally invasive approach for uncomplicated PDH with rapid postoperative recovery. Early clinical intervention is pivotal for PDH prognosis, as delayed treatment may trigger severe complications including intestinal strangulation and necrosis; even asymptomatic PDH warrants elective surgery to eliminate potential risks. With the progress of minimally invasive surgical technologies, PDH treatment will become more precise and secure.

Overall, improved PDH awareness, timely imaging-based diagnosis and standardized surgical selection are key to optimizing patient prognosis. Most notably, this study provides critical guidance for emergency surgeons: PDH must be prioritized in the differential diagnosis of unexplained small bowel obstruction in young adults. Active vigilance and targeted imaging evaluation for suspected PDH in emergent settings can prevent diagnostic delays, enable timely minimally invasive intervention, and effectively reduce the incidence of fatal intestinal complications, which is of great clinical significance for managing unexplained acute small bowel obstruction in young patients.

## Data Availability

The original contributions presented in the study are included in the article/Supplementary Material, further inquiries can be directed to the corresponding author.
